# Forskolin Induces Endocrine Disturbance in Human JEG-3 Placental Cells

**DOI:** 10.3390/toxics10070355

**Published:** 2022-06-30

**Authors:** Patrice Rat, Pascale Leproux, Sophie Fouyet, Elodie Olivier

**Affiliations:** 1 Faculty of Pharmaceutical Sciences and Biology, Université Paris Cité, CNRS, CiTCoM, 75006 Paris, France; patrice.rat@u-paris.fr (P.R.); pascale.leproux@u-paris.fr (P.L.); sophie.fouyet@etu.u-paris.fr (S.F.); 2 Léa Nature, 17180 Périgny, France

**Keywords:** placenta, forskolin, endocrine disruptors, P2X7 receptor, hormones

## Abstract

Forskolin, used in folk medicine since ancient times, is now available as a dietary supplement, with an indication as a fat burner and appetite suppressant. However, the safety of forskolin is poorly documented especially for pregnant women. The question that we raised is what about the safety of forskolin in pregnant women? As the placenta, an endocrine organ, is the key organ of pregnancy, we evaluated the in vitro placental toxicity of forskolin. We focused first on the activation of a P2X7 degenerative receptor as a key biomarker for placental toxicity, and second on steroid and peptide hormonal secretion. We observed that forskolin activated P2X7 receptors and disturbed estradiol, progesterone, hPL and hyperglycosylated hCG secretion in human placental JEG-Tox cells. To the best of our knowledge, we highlighted, for the first time, that forskolin induced endocrine disturbance in placental cells. Forskolin does not appear to be a safe product for pregnant women and restrictions should be taken.

## 1. Introduction

Forskolin, a diterpenoid isolated from the *Lamiaceae Coleus forskohlii* has been used since ancient times in Indian folk medicine for the treatment of heart diseases, abdominal colic, respiratory disorders such as asthma and topical disorders such as eczema [1]. Uses of this plant for similar treatments have also been reported in Africa and South America [1,2]. More recently, forskolin has been used in glaucoma treatment and has been identified as a promising molecule in cancer therapy especially for acute leukemia and triple negative breast cancer [3,4,5,6,7,8,9]. Forskolin is also used for the management of weight and the metabolic risk associated in overweight people [10,11]. For the indications of burning fat and being an appetite suppressant, forskolin is commercially available, generally as a dietary supplement in capsules over-the-counter in pharmacies and online. Consequently, forskolin can be easily bought without any medical advice and without any restrictions or prevention messages, apart for people suffering from ulcers, since the work of Hersey’s et al. showing that forskolin may increase stomach acid levels [12]. The safety of forskolin is poorly documented, in particular in pregnant women. In the literature, Shu et al. showed that forskolin delayed spontaneous meiotic progression in immature human oocytes [13] and Almeida and Lemonica showed a delayed fetal development and an anti-implantation effect in rats of a natural extract containing forskolin used in folk medicine as an abortifacient [14].

The question that we raised is what about the safety of forskolin in pregnant women?

The intake of drugs including over-the-counter and natural supplements can be dangerous during pregnancy. Indeed, pregnancy is a sensitive period and these products can induce adverse effects not only for the mother but also for the unborn child.

The placenta is a key organ of pregnancy as it supports the normal growth and development of the fetus. Indeed, the placenta coordinates gas exchanges, metabolic transfer, immunological functions and has an endocrine function as it produces, metabolizes and regulates numerous hormones including polypeptide and steroid hormones [15,16]. The endocrine function of the placenta is crucial for the pregnancy process and the growth of the fetus. As a consequence, any alteration in the level of steroid and polypeptide hormones such as estradiol, progesterone, human placental lactogen (hPL) or human chorionic gonadotropin (hCG) is associated with adverse pregnancy outcomes such as preeclampsia, intrauterine growth restriction and preterm birth [17,18,19,20,21,22]. Preeclampsia and preterm birth have also been associated with the P2X7 degenerative purinoreceptor [23,24]. In our previous studies, we observed that the activation of the P2X7 membrane receptor is a marker of placental toxicity including endocrine-disrupting chemical-induced toxicity. Indeed, we highlighted the P2X7 receptor activation as a common mechanism of many endocrine-disrupting chemicals and suspected endocrine disrupting chemicals, including bisphenol A and diethylstilbestrol, in human placental cells [25,26,27].

The aim of this study is to evaluate in vitro placental toxicity of forskolin. To this purpose, we incubated human placental JEG-Tox cells with forskolin as we previously demonstrated that JEG-Tox cells can be of great value in placental toxicology studies [28]. We evaluated P2X7 receptor activation and hormonal secretion of estradiol, progesterone, hPL and hyperglycosylated hCG.

## 2. Materials and Methods

### 2.1. Chemicals and Reagents

Forskolin, from *Coleus forskohlii*, ≥98% (HPLC) was purchased from Merck (Darmstadt, Germany). A 100 mM stock solution in dimethylsulfoxyde (DMSO, PanReac, Barcelona, Spain) was prepared and stored at −20 °C. Dilutions in culture medium were prepared just before the cell incubation. The same final concentration of DMSO on cells was used for all concentrations of forskolin, i.e., 0.1%.

Cell culture reagents: MEM medium, phosphate buffered saline (PBS), trypsin, fetal bovine serum (FBS), penicillin-streptomycin and glutamine were purchased from Gibco Life Technologies (Paisley, UK). Flasks and 96-well microplates were purchased from Corning (Amsterdam, The Nederlands).

A YO-PRO-1 (cat. no. Y3603) fluorescent probe was obtained from ThermoFisher Scientific.

Fluorescence Resonance Energy Transfer (FRET, cat. no. 62ESTPEG and 6FPROPEG) and ELISA (cat. no. MBS705577 and MBS764095) kits were purchased from Cisbio Biosassays (Codolet, France) and MyBioSource (Vancouver, BC, Canada), respectively.

### 2.2. Cell Line and Culture Condition

The choriocarcinoma-derived JEG-3 cell-line (ATCC HTB-36, Manassas, VA, USA), was selected based on the results of previous studies highlighting the use of these endocrine cells for placental toxicity studies [25,26,27,28]. JEG-3 cells were cultured in Minimum Essential Medium Eagle’s medium supplemented with 10% FBS, 2 mM of glutamine, 50 U/mL of penicillin and 50 µg/mL of streptomycin. Cells were detached using trypsin, counted, and then seeded at 80,000 cells/mL in 96-well microplates (200 μL by well). The cells were incubated for 72 h with a concentration range of forskolin from 0.0001 to 100 µM in MEM supplemented with 2.5% FBS according to Olivier et al.’s protocol that describes the JEG-Tox model [28].

### 2.3. Cell Viability Evaluation

The 0.1 mg/mL alamar blue solution was diluted in MEM supplemented with 2.5% FBS to obtain a working concentration of 0.009 mg/mL. The alamar blue working solution was distributed in each well (200 µL/well) for a 6-h incubation time at 37 °C. Then, the fluorescence signal was read (λ_ex_ = 535 nm, λ_em_ = 600 nm) using a microplate reader (Spark, Tecan).

### 2.4. P2X7 Receptor Activation

The YO-PRO-1^TM^ fluorogenic probe only enters into cells after the P2X7 receptor activation-induced pore opening and binds to DNA emitting fluorescence. A 1 mM YO-PRO-1 stock solution was diluted at 1/500 in PBS just before use to obtain a 2 µM working solution. The YO-PRO-1 working solution was distributed in each well (100 μL/well) following our validated protocol [29]. After a 10-min incubation period at room temperature away from light, the fluorescence signal was read (λ_ex_ = 485 nm, λ_em_ = 531 nm) using a microplate reader (Spark, Tecan).

### 2.5. Hormonal Dosages

After a 72-h incubation period in forskolin diluted in cell culture medium supplemented with 2.5% FBS, the cells were centrifuged, and cell supernatants were collected.

Estradiol and progesterone were quantified in cell supernatants by FRET technology (HTRF Cisbio Biosassays, Codolet, France) according to manufacturer’s instructions. Human placental lactogen (hPL) hormone and human hyperglycosylated Chorionic Gonadotropin (hCG) hormone were measured by sandwich ELISA (MyBioSource) according to manufacturer’s instructions.

### 2.6. Statistical Analysis

The means of at least four independent experiments were calculated and normalized to culture medium. Results are expressed in percentage (for cell viability) or fold change (for P2X7 receptor activation and hormonal quantitation) compared with culture medium and presented as means of at least three independent experiments ± standard error of the means. After a D’Agostino–Pearson omnibus normality test to check the normal distribution of the data, a one-way ANOVA followed by a Dunnett’s test were performed (α risk = 5%) using GraphPad Prism 8 software (San Diego, CA, USA). Thresholds of significance were * *p* < 0.05, ** *p* < 0.01, *** *p* < 0.001 and **** *p* < 0.0001 compared to the culture medium. In all the experiments, the culture medium and control solvent were not statistically different.

## 3. Results

### 3.1. Cell Viability

**Figure 1:**
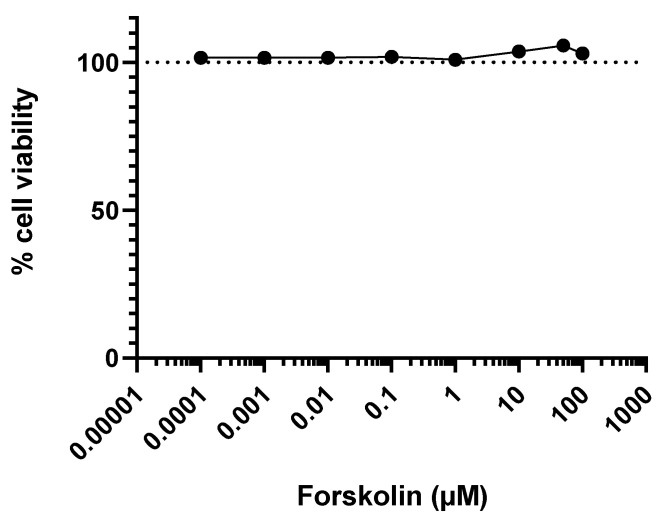
Cell viability evaluation using the alamar blue assay after a 72-h incubation time with forskolin.

The cell viability was evaluated in JEG-Tox cells after a 72-h incubation time using the alamar blue assay (Figure 1). Forskolin did not induce any loss in cell viability.

### 3.2. P2X7 Activation

**Figure 2:**
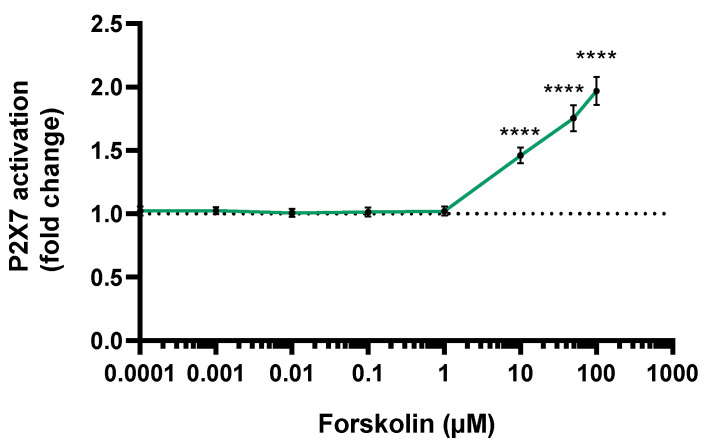
P2X7 receptor activation using the YO-PRO-1 assay after a 72-h incubation time with forskolin. The dotted line represents the P2X7 receptor activation basal level. The significance threshold was **** *p* < 0.0001 compared to the culture medium.

The P2X7 receptor activation was evaluated in JEG-Tox cells after a 72-h incubation time using the YO-PRO-1 assay. Forskolin activated the P2X7 receptor in a dose-dependent manner (Figure 2). The P2X7 receptor was statistically significantly activated by forskolin from 10 µM: ×1.5 at 10 µM, ×1.79 at 50 µM and ×2.15 at 100 µM.

### 3.3. Estradiol Secretion

**Figure 3:**
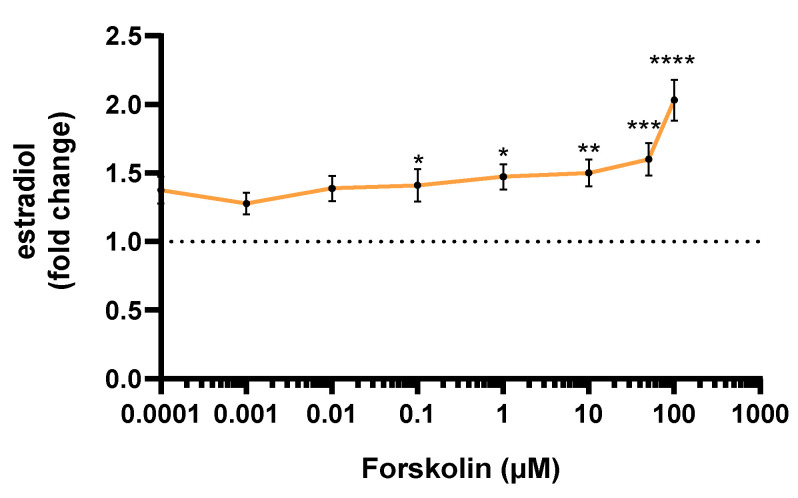
Estradiol secretion by JEG-Tox cells after a 72-h incubation time with forskolin. The dotted line represents the estradiol basal level. The significance thresholds were * *p* < 0.05, ** *p* < 0.01, *** *p* < 0.001 and **** *p* < 0.0001 compared to the culture medium.

The estradiol secretion by JEG-Tox cells after a 72-h incubation time was quantified in cell supernatants. Forskolin induced a statistically significant raise in estradiol secretion from 0.1 µM: ×1.41 at 0.1 µM, ×1.47 at 1 µM, ×1.50 at 10 µM, ×1.60 at 50 µM and ×2.03 at 100 µM (Figure 3).

### 3.4. Progesterone Secretion

**Figure 4:**
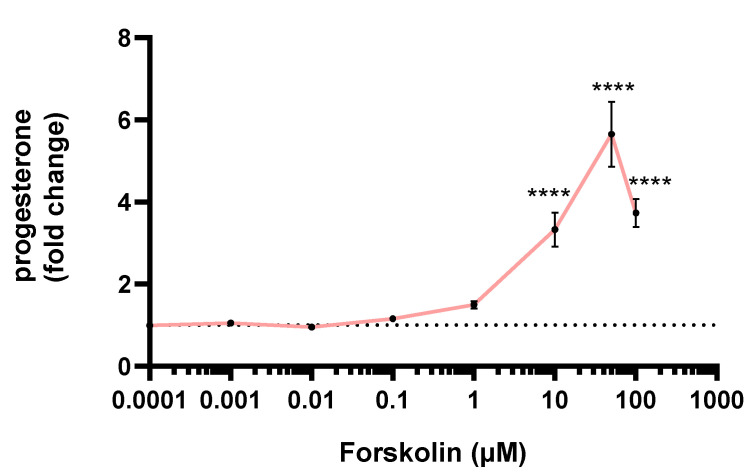
Progesterone secretion after a 72-h incubation time with forskolin. The dotted line represents the progesterone basal secretion. The significance threshold was **** *p* < 0.0001 compared to the culture medium.

The progesterone secretion by JEG-Tox cells after a 72-h incubation time was quantified in cell supernatants. Forskolin induced a statistically significant raise in progesterone secretion from 10 µM: ×3.33 at 10 µM, ×5.65 at 50 µM and ×3.73 at 100 µM (Figure 4).

### 3.5. hPL Secretion

**Figure 5:**
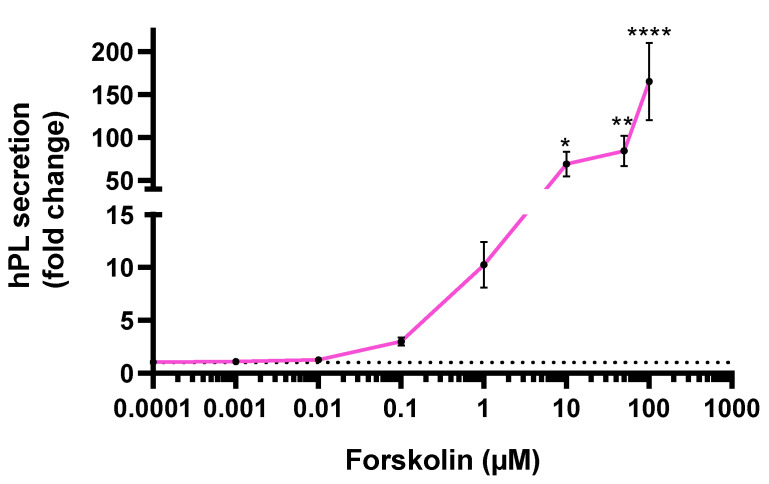
hPL secretion after a 72-h incubation time with forskolin. The dotted line represents the hPL basal secretion. The significance thresholds were * *p* < 0.05, ** *p* < 0.01 and **** *p* < 0.0001 compared to the culture medium.

The hPL secretion by JEG-Tox cells after a 72-h incubation time was quantified in cell supernatants (Figure 5). Forskolin induced a statistically significant raise in hPL secretion from 10 µM: ×69.21 at 10 µM, ×84.65 at 50 µM and ×165.4 at 100 µM.

### 3.6. Hyperglycosylated hCG Secretion

**Figure 6:**
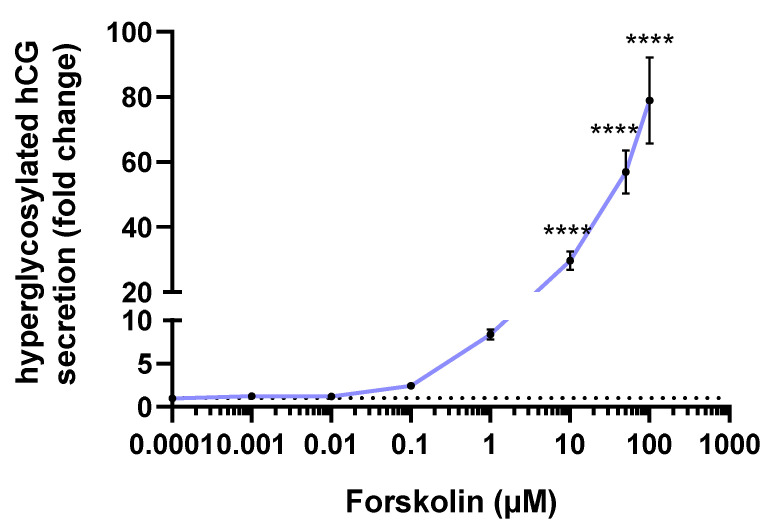
Hyperglycosylated hCG secretion after a 72-h incubation time with forskolin. The dotted line represents the hyperglycosylated hCG basal secretion. The significance threshold was **** *p* < 0.0001 compared to the culture medium.

The hyperglycosylated hCG secretion by JEG-Tox cells after a 72-h incubation time was quantified in cell supernatants (Figure 6). Forskolin induced a dose-dependent increase in hyperglycosylated hCG release. This raise was statistically significant from 10 µM: ×29.61 at 10 µM, ×56.91 at 50 µM and ×78.96 at 100 µM.

## 4. Discussion

The objective of the present study was to assess the potential placental toxicity of forskolin in vitro. Forskolin is available, without any medical advice, over-the-counter in pharmacies and as natural supplements sold online principally as a fat burner or appetite suppressant. Pregnant women concerned about limiting their weight gain may be tempted to try these natural products, but natural does not mean safe. Natural supplements containing forskolin generally recommend an intake of 50 mg of forskolin a day, which represents 122 µM forskolin daily. Using SwissADME software, the log P_o/w_ is 2.67, meaning a high probability of absorption and consequently the gastrointestinal absorption of forskolin is high. The tested concentrations seem to be in the same order as this intake, especially as it has been demonstrated that chemicals are more concentrated in the placenta than in maternal tissues [28].

The placenta plays a key role during pregnancy, supporting the normal growth and development of the fetus. Due to this key role, the placenta can be considered as a target for toxic agents. Indeed, any alteration of the placenta can induce many pregnancy short-term outcomes such as preeclampsia and intrauterine growth restriction [19,20,21] and long-term outcomes such as cognitive and visual development during childhood [30,31]. The P2X7 receptor has been associated with short-term pregnancy disorders such as preeclampsia and preterm birth [23,24] and to long-term disorders of the offspring such as autism-like spectrum [32].

To perform our study, we selected a human placental model, the JEG-3 cells, as we previously demonstrated that JEG-Tox cells can be of great value in placental toxicology studies [28]. We demonstrated in our previous work in these cells that the activation of the P2X7 receptor is a marker of placental toxicity including endocrine-disrupting chemical-induced toxicity, as we highlighted that the well-known endocrine-disrupting chemicals known as bisphenol A or diethylstilbestrol, despite their chemical differences, shared a common mechanism: the activation of the P2X7 degenerative receptor in human placental cells [25,26,27]. Our results showed an activation of the P2X7 degenerative receptor induced by non-cytotoxic concentrations of forskolin from 1µM, demonstrating a placental toxicity of forskolin.

The key role played by the placenta during pregnancy is also based on its endocrine function as the placenta produces, metabolizes and regulates numerous hormones including steroid and polypeptide hormones, such as estradiol, progesterone, human placental lactogen (hPL) or human chorionic gonadotropin (hCG) and its hyperglysosylated isoform [16]. Any alteration in these hormone levels, whether it is excessively or deficiently released, can have an impact on fetal development and pregnancy maintenance and also on the mother’s health, as preeclampsia, gestational diabetes, intrauterine growth restriction, preterm birth and miscarriages are associated to altered levels of steroid and polypeptide hormones [17,18,33,34].

Our results showed that forskolin disturbed placental hormone secretion of human JEG-3 cells. Indeed, forskolin had a stimulatory effect on estradiol secretion. Estrogens are synthesized exclusively in the placenta during pregnancy and play a key role in fetal development as they promote placental angiogenesis and uterine artery vasodilatation [35]. The estrogen synthesis from C19 steroids during placental steroidogenesis is regulated by a key enzyme: the aromatase cytochrome P450. Harada et al. have shown that forskolin increases aromatase activity and mRNA levels in JEG-3 cells, which can explain the stimulatory effect on estradiol secretion that we observed [36]. Forskolin exerts its effects mainly by stimulating adenylate cyclase, which in consequence increases the cellular concentration of the second messenger cyclic AMP (cAMP). Aromatase protein expression and activity are controlled by cAMP-dependent intracellular signal pathways [37,38]; that is why the increase in estradiol secretion induced by forskolin could be associated, at least in part, with its increase in cAMP, which in turn increases the aromatase activity. In clinics, high estradiol levels are related to low birth weight and small-for-gestational-age birth, and newborn dyslipidemia, which can be related to dyslipidemia during adulthood [39,40].

Placental estradiol level can impact progesterone levels as several studies have shown that the addition of estradiol in human trophoblasts in culture or in ex vivo placental explants increased the production of progesterone [41,42,43]. The stimulatory effect on progesterone secretion induced by forskolin observed herein can be associated with the increased level of estradiol. Moreover, this increase in progesterone levels can also be related to the cAMP level increase due to forskolin, as cAMP increases the secretion of progesterone [44,45]. In clinics, high levels of progesterone during pregnancy is associated with gestational diabetes [33,46], which increases the risk of pre-term birth, preeclampsia, macrosomia and perinatal mortality [47].

The human placental lactogen (hPL) and hyperglysosylated human chorionic gonadotropin (hCG) hormones are specific to pregnancy. Any disturbance in these peptide hormone levels are associated with adverse pregnancy outcomes. Indeed, hPL plays a metabolic role, mobilizing fatty acids from the mother and acting as an insulin antagonist to provide glucose to the fetus [48]. Alteration of hPL levels is associated, as a consequence, with growth retardation in the case of a decrease, gestational diabetes or fetal macrosomia depending on the increase of its level [49]. Gestational diabetes and fetal macrosomia are associated with a higher risk of pregnancy complications including preterm birth, preeclampsia and perinatal mortality [47]. Hyperglycosylated hCG isoform plays a key role during embryo implantation and early fetal development as an invasion promoter [50]. We focused on hyperglysocylated hCG as it plays a pivotal role to prevent early pregnancy loss, spontaneous abortion and preeclampsia [51]. JEG-3 cells produced predominantly hyperglycosylated hCG [52]. Alteration of hyperglycosylated hCG levels is associated with spontaneous abortion, preeclampsia (in case of decrease of this level), placenta acreta, Down’s syndrome, and is considered as a tumor marker for gestational trophoblastic diseases (in case of increase of this level), as observed herein with forskolin [51,53,54,55].

Taken together, our results showed a forskolin-induced alteration of a placental toxicity marker: the P2X7 degenerative receptor and an alteration of the endocrine function of the placenta as previously observed with numerous endocrine-disrupting chemicals. As a consequence, forskolin has a similar profile as endocrine-disrupting chemicals and could present a risk during pregnancy, leading to adverse pregnancy outcomes such as gestational diabetes, preterm birth or preeclampsia. Forskolin endocrine effects have also been assessed in the human adrenal cortex-derived cell line H295R. In these cells, forskolin induces and stimulates steroidogenesis and consequently increases estradiol and testosterone levels [56].

## 5. Conclusions

To the best of our knowledge, this study shows, for the first time, the impact of forskolin on placental human cells. Forskolin induced the P2X7 degenerative receptor activation and disturbed steroid (estradiol and progesterone) and polypeptide (hPL and hyperglycosylated hCG) hormone secretions. These results completed the endocrine effects induced by forskolin observed in other human cells and the delayed fetal development observed in an in vivo study. Taken together, all these data highlighted that forskolin may induce adverse effects for pregnant women and their offspring; pregnant women should be advised not to use this product, although direct human evidence is not available.

## Data Availability

Not applicable.
